# Advances of Nanotechnology in the Diagnosis and Treatment of Hepatocellular Carcinoma

**DOI:** 10.3390/jcm12216867

**Published:** 2023-10-31

**Authors:** Rebeca Escutia-Gutiérrez, Ana Sandoval-Rodríguez, Adalberto Zamudio-Ojeda, Santiago José Guevara-Martínez, Juan Armendáriz-Borunda

**Affiliations:** 1 Department of Molecular Biology and Genomics, Institute for Molecular Biology in Medicine and Gene Therapy, Health Sciences University Center, University of Guadalajara, Guadalajara 44340, Mexico; rebeca.escutia@academicos.udg.mx (R.E.-G.); anasol44@hotmail.com (A.S.-R.); 2 Department of Physics, Exact Sciences and Engineering University Center, University of Guadalajara, Guadalajara 44340, Mexico; adalberto.zojeda@academicos.udg.mx; 3 Tecnologico de Monterrey, School of Medicine and Health Sciences, Zapopan 45201, Mexico

**Keywords:** hepatocellular carcinoma, nanoparticles, theragnostic, drug delivery

## Abstract

Nanotechnology has emerged as a promising technology in the field of hepatocellular carcinoma (HCC), specifically in the implementation of diagnosis and treatment strategies. Nanotechnology-based approaches, such as nanoparticle-based contrast agents and nanoscale imaging techniques, have shown great potential for enhancing the sensitivity and specificity of HCC detection. These approaches provide high-resolution imaging and allow for the detection of molecular markers and alterations in cellular morphology associated with HCC. In terms of treatment, nanotechnology has revolutionized HCC therapy by enabling targeted drug delivery, enhancing therapeutic efficacy, and minimizing off-target effects. Nanoparticle-based drug carriers can be functionalized with ligands specific to HCC cells, allowing for selective accumulation of therapeutic agents at the tumor site. Furthermore, nanotechnology can facilitate combination therapy by co-encapsulating multiple drugs within a single nanoparticle, allowing for synergistic effects and overcoming drug resistance. This review aims to provide an overview of recent advances in nanotechnology-based approaches for the diagnosis and treatment of HCC. Further research is needed to optimize the design and functionality of nanoparticles, improve their biocompatibility and stability, and evaluate their long-term safety and efficacy. Nonetheless, the integration of nanotechnology in HCC management holds great promise and may lead to improved patient outcomes in the future.

## 1. Introduction

Hepatocellular carcinoma (HCC) is the most common type of liver cancer and one of the top ten leading causes of cancer-related deaths worldwide [1]. HCC development is highly associated with liver fibrosis and cirrhosis [2]. The main etiologies include viral hepatitis (B or C virus), alcoholic liver disease, and metabolic syndrome [3]. HCC patients have a 20% 5-year survival rate [4,5]. HCC diagnosis includes ultrasound, computed tomography, or magnetic resonance imaging and biomarker quantification, such as alpha-fetoprotein (AFP) [6,7].

HCC treatment includes resection surgery and liver transplantation, indicated in a very small number of patients, while systemic chemotherapy has moderate results due to high recurrence and multidrug resistance. Treatments like radiofrequency ablation (RFA) or transarterial chemoembolization (TACE) have only a palliative role [7,8]. At the cellular level, chronic liver damage provokes liver sinusoidal endothelial cells (LSECs) injury, known as capillarization, in which crosstalk between hepatic parenchyma and hepatic artery and portal veins is affected, leading to fibrosis through hepatic stellate cells (HSCs) activation [9]. HSC activation triggers key molecular fibrogenic responses, like TGF beta upper expression, and in consequence, increased collagen deposition in the extracellular matrix (ECM) is observed. This fibrotic microenvironment progressively leads to carcinogenesis through the upregulation of growth factors like epidermal growth factor (EGF), fibroblast growth factor (FGF), hepatocyte growth factor (HGF), platelet-derived growth factor (PDGF), and vascular endothelial growth factor (VEGF), which facilitate the survival and proliferation of HCC cells [10]. The suppression of the pathways that involve these receptors and the molecules involved in oxidative liver damage and fibrosis are some of the possible therapeutic targets for HCC, besides their diagnosis applications, that can be encompassed from a nanomedicine perspective.

Nanomaterial shapes include spheres, sheets, and tubes between 1 and 100 nm, and their biocompatible and biodegradable properties vary according to composition, which may include inorganic materials: metals, metal oxides, inorganic salts, and silica; carbon-based nanoparticles (NPs): comprise polymeric NPs, micelles, liposomes, lipid NPs, and carbon dots (CDs). Nanomaterials with specialized structures include upconversion NPs, metal–organic frameworks (MOFs), and Janus NPs (an NP whose surface has distinct regions with differing physical properties) [11,12]. When developed as a drug delivery system (DDS), nanoparticles are desired to reach a specific organ or area. Targeting can be passive when NPs are usually coated with polyethylene glycol (PEG) to reduce their recognition as a foreign molecule by the body’s immune system. Consequently, they are kept in the bloodstream long enough to reach the desired location. Also, when desired, targeting can be active by incorporating some molecules that contribute to a specific affinity for the target cell. Some examples of active targeting are the incorporation of folic acid, peptide conjugation, asialoglycoprotein receptor, lactose conjugation, etc. [13]. Nanomedicine has had robust development in the last two decades. Due to the still existing difficulties in HCC detection at early stages and the poor prognoses when diagnosed, the aim of this review is to extensively describe the current efforts in the nanomaterials field and to show the most promising strategies for the treatment and diagnosis of HCC.

## 2. Hepatocellular Carcinoma

### 2.1. Epidemiology and Risk Factors

According to the Global Cancer Observatory, HCC is the second most common cancer on the globe and the fifth most common cancer overall [14]. By 2025, hepatocellular carcinomas (HCC), which account for 80% of all liver malignancies, will affect more than a million people annually [6]. The most important risk factor for HCC is cirrhosis, regardless of the cause [15]. HCC has a poor overall survival rate, with a mean survival of 6–10 months.

Several important risk factors are linked to HCC, such as hepatitis B virus (HBV) and hepatitis C virus (HCV) infection, non-alcoholic fatty liver disease, increased age, male gender, and Asian ethnicity, along with the severity and activity of inflammation and fibrosis. Metabolic risk factors like diabetes and obesity and behavioral risk factors such as alcohol intake and smoking also play a role in the development of HCC [16,17].

### 2.2. Physiopathology

Hypervascular tumors are the hallmark of HCC, and angiogenesis is crucial to the initiation, growth, progression, and eventual illness dissemination to other organs [18].

HCC develops in healthy hepatocytes over time as a result of the progressive accumulation of essential molecular changes that endow them with cancer-specific capacities. Under the continual pressure of clonal selection, specific subclones of cells with an advantage in growth and survival will dynamically go through clonal expansion. In cirrhotic livers, persistent inflammation and fibrinogenesis predispose the liver to dysplasia and malignant transformation [18,19].

Hepatic stem cells with the potential to proliferate in response to HCV/HBV cell damage induce chronic re-generation that may give rise to HCC [20]. Even without cirrhosis, HBV can result in HCC due to genomic instability and chromosomal rearrangements caused by HBV’s DNA integration into the host genome and the expression of HBV-encoded X protein, which is known to alter tumor suppressor genes and oncogenes. HCV-related carcinogenesis results from a complex combination of host, environmental, and viral factors. Mainly, HCV dysregulates host lipid metabolism, causing liver fat accumulation. HCV is also able to induce angiogenic and metastatic pathways, and malignant transformation of hepatocytes occurs through increased liver cell turnover [21].

### 2.3. Standard Diagnosis

The American Association for the Study of Liver Diseases (AASLD) and the European Association for the Study of the Liver (EASL) advise that the diagnosis of HCC should be based on noninvasive imaging criteria and/or pathology examination [22]. The noninvasive diagnosis of HCC should be based on either dynamic contrast-enhanced MRI or multiphasic CT, while the pathological diagnosis of HCC should be based on the definitions of the International Consensus Group for Hepatocellular Neoplasia. Alpha-fetoprotein and other blood indicators often play a little part in the diagnosis of HCC [8,23]. The LI-RADS (Liver Imaging Reporting and Data System) categorizes observations such as lesions or pseudolesions > 10 mm visible on multiphase exams based on their likelihood of being benign, hepatocellular carcinoma, or another hepatic malignant neoplasm such as cholangiocarcinoma or combined HCC-cholangiocarcinoma. Both LI-RADS-1 and LI-RADS-2 indicate benign conditions [24].

### 2.4. Standard Treatment

#### 2.4.1. Therapy in Early Stages

International recommendations showed modest variations in the suggested therapeutic strategy between Asia, Europe, and the United States [25].

Early-stage HCC is defined as a compensated liver function (Child-Pugh A or B) and a limited tumor burden (one small tumor or three tumors no larger than 3 cm). Patients who meet these criteria typically receive curative care and have an excellent prognosis [26].

The primary curative option for hepatocellular cancer patients is surgery (liver resection or liver transplantation). Those with isolated tumors and preserved liver function are the best candidates for liver surgery. Patients with multifocal disease or decompensated cirrhosis are typically advised to undergo a liver transplant. Patients with cirrhosis who develop hepatocellular carcinoma require sophisticated surgical care. Therefore, multidisciplinary teams in seasoned centers should evaluate patients and consider both resection and transplantation [27]. The primary restriction to liver transplantation is organ availability. Patients are chosen for liver transplantation based on strict selection criteria that take into account the size and number of tumors [28].

Thermal ablation is advised for patients with early-stage hepatocellular carcinoma, as well as for individuals with 2–4 cm tumors that are ineligible for surgical excision due to anatomical reasons or patient circumstances. Radiofrequency ablation (RFA) is the most commonly utilized ablation technique for hepatocellular cancer treatment. The procedure involves inflicting thermal damage on tumor tissue via electromagnetic radiation deposition. Local ablation is now regarded as a potentially curative therapy for small hepatocellular carcinomas (approximately three centimeters), and most guidelines advocate RFA as first-line therapy for solitary tumors smaller than two centimeters [29].

A new technique for eliminating tumors is irreversible electroporation (IRE). It uses non-thermal, high-voltage electric pulses to hit tumor tissue. By accomplishing this, it makes the cell membrane more permeable, upsetting cellular homeostasis and inducing apoptosis. The lack of the “heat sink” effect and the ability to treat nearby veins, bile ducts, and other important structures are two advantages of IRE versus RFA [30].

Because hepatocellular carcinoma tumors are hypervascular and receive the majority of their blood supply from the hepatic artery, intra-arterial therapy is a critical component of treatment for intermediate-stage hepatocellular carcinoma. Intra-arterial treatment consists of the selective catheter infusion of particles (with or without chemotherapeutic drugs) directed to the arterial branch of the hepatic artery, feeding the portion of the liver in which the tumor is located [31].

There are three different methods of intra-arterial therapy: radioembolization, bland particle embolization (TAE), and chemoembolization (also known as conventional trans-arterial chemoembolization or drug-eluting bead-TACE). The basis of TAE is to decrease or eliminate blood flow to the tumor, resulting in tumor ischemia and necrosis. At the same time, TACE focuses on reducing chemotherapy-induced systemic toxicity while delivering near-tumor cells a highly concentrated dosage of chemotherapy and extending the duration of chemotherapy exposition.

Radiation therapy (RT) delivers high-dose radiation to liver tumors while preserving adjacent liver tissue, reducing the risk of radiation-induced liver damage in patients with unresectable or inoperable HCC. Stereotactic body radiation (SBRT), proton treatment, and interstitial brachytherapy are the radiotherapy methods most frequently used for hepatocellular cancer. These methods’ large tumor doses are possible while lowering the chance of radiation-induced liver damage thanks to their local precision. One-year local control rates of SBRT in several small prospective studies performed on hepatocellular cancer ranged from 75 to 95%. A total of 144 major cohort studies and meta-analyses of retrospective investigations, including individuals with early hepatocellular carcinoma and those at high risk for portal vein infiltration, support these findings [32].

#### 2.4.2. Systemic Therapy in Advanced Stages

Most patients, when diagnosed with HCC, have an advanced stage, and only a small fraction are candidates for curative therapy. Furthermore, with a large variety of local therapies available to treat patients with unresectable HCC limited to the liver, systemic therapy has frequently been reserved as a last choice for those at a very advanced stage. Until recently, the only systemic treatment option for people with advanced illnesses was so rare. However, several new systemic therapy alternatives for the initial treatment of advanced HCC and a number of active drugs for HCC during or after earlier systemic treatment have emerged from a number of recent clinical trials [33].

Since 2017, there has been a notable improvement in the survival rate of individuals taking systemic drugs. Sequential therapy should be frequently taken into consideration for patients with advanced hepatocellular carcinoma; eight regimens have been approved by the US Food and Drug Administration (FDA) and six of them by the European Medicines Agency (EMA) [34].

#### First Line Treatments

In patients with advanced hepatocellular cancer, sorafenib was the first targeted therapy to demonstrate efficacy. Sorafenib is a multikinase inhibitor for RAF, platelet-derived growth factor receptors, VEGF receptors, and a number of other tyrosine kinases [35,36]. Sorafenib significantly improves patient survival rates. Even though sorafenib is now commonly used, it still has certain unfavorable side effects. For instance, sorafenib can induce skin rash, diarrhea, high blood pressure, and redness in the palms or soles of the feet due to sorafenib’s non-specific absorption by normal tissues [37].

Lenvatinib is an oral tyrosine kinase inhibitor (TKI) with action against VEGFR1-3 (vascular endothelial growth factor receptor 1-3), FGFR1-4 (fibroblast growth factor receptor 1-4), PDGF (platelet-derived growth factor), RET (proto-oncogene tyrosine-protein kinase receptor), and KIT (proto-oncogene tyrosine-protein kinase kit). Lenvatinib was found to be non-inferior to sorafenib in the first-line setting in the phase III REFLECT investigation, which largely included Asian patients. The mean overall survival was 136 months in the lenvatinib arm vs. 123 months in the sorafenib arm. In the primary and secondary outcomes of median progression-free survival and overall response rate, lenvatinib surpassed sorafenib [38].

Immune-checkpoint inhibitors (ICIs) are a type of cancer immunotherapy, like PD-1 or PD-L1 inhibitors; these are important regulators of antitumor T cell responses. PD-1, CTLA4, LAG3, and TIM3 are examples of immune-checkpoint receptors that naturally regulate T cell activity in maintaining self-tolerance, while CD28, GITR, and OX40 are examples of the costimulatory immune-checkpoint proteins that promote T cell expansion [39]. Monotherapy with ICIs has shown clinically substantial benefits [40], and combining ICIs with anti-angiogenic drugs, as well as the merging of two independent ICIs, are being studied in larger patient groups. In 2020, atezolizumab (ATZ), in combination with bevacizumab (BVZ), was approved by the U.S. Food and Drug Administration (FDA) for unresectable hepatocellular carcinoma treatment. Atezolizumab is an immune checkpoint inhibitor that acts by blocking PD-L1 (anti-programmed death-1), an inhibitory receptor expressed on activated T cells under conditions of prolonged stimulation, such as infection or cancer [41]. Bevacizumab is a monoclonal antibody that targets vascular endothelial growth factor (VEGF) [42].

More recently, the Phase III IMbrave150 trial reported a hazard ratio (HR) for overall survival (OS) of 0.58 in favor of ATZ and BVZ, representing a 42% reduction in the risk of death compared to sorafenib, previously recommended as the first-line therapy for patients with HCC [42,43].

Other ICIs, like durvalumab in combination with tremelimumab, are being explored for patients with unresectable hepatocellular carcinoma (HCC). The STRIDE regimen, which is composed of a single dose of tremelimumab paired with durvalumab, met the primary endpoint of statistically significant improvement in overall survival versus sorafenib in patients. According to HIMALAYA’s findings, a single dosage of tremelimumab is sufficient to add clinical efficacy to durvalumab monotherapy [44].

##### Second-Line Treatments

Roughly 50% of HCC patients receive systemic therapies, which are frequently given as sorafenib or lenvatinib as first-line treatments and regorafenib or cabozantinib as second-line treatments. Regorafenib is a fluorinated sorafenib analog that has a similar molecular target profile. The RESORCE phase III trial was the first to show promising results in the second-line setting for patients with advanced hepatocellular cancer who had failed sorafenib. The trial’s primary goal was attained when it demonstrated that regorafenib outperformed placebo in terms of mean overall survival [45].

Both the EMA and the FDA have approved cabozantinib, a tyrosine kinase inhibitor with activity against many targets, including MET, VEGFR, and the TAM kinase family (TYRO-3, AXL, and MER), as a second-line treatment for patients with advanced hepatocellular carcinoma [36].

Anti-programmed death-1 (PD-1) monoclonal antibodies like pembrolizumab and nivolumab suppress immune checkpoint signaling. Nivolumab’s effectiveness and safety as a second-line therapy for HCC were confirmed in the CheckMate-040 trial [46]. Based on the findings of the CheckMate-040 research, nivolumab has been granted medical licenses in many nations for the treatment of sorafenib-treated HCC patients [47].

Ramucirumab is an anti-angiogenic monoclonal antibody that has progressed to clinical studies in HCC. Ramucirumab acts by reducing the activation of VEGFR2 (vascular endothelial growth factor receptor 2). Ramucirumab was approved for the treatment of HCC by the FDA as a result of the major Phase 3 clinical trial known as the REACH-2 trial. Patients with advanced HCC who had previously taken sorafenib but had suffered disease progression were the subjects of the study. In comparison to placebo, ramucirumab increased overall survival. Additionally, ramucirumab is frequently used in combination with another immune checkpoint inhibitor called atezolizumab [48,49].

## 3. Advances in Applications of Nanotechnology in HCC

Nowadays, the use of nanomaterials in various technological and research areas is becoming more common. This is motivated by the properties of materials at nanometric scales, which are different from those observed at the macroscopic level. In general, the properties of nanoparticles depend on the material they are made of, as well as the shape, size, and arrangement of the atoms. However, the property that nanoparticles, in general, have in common is the high ratio of surface atoms to bulk atoms, as well as their dimensions ranging from 1 to 100 nm. Some examples of the properties presented by nanoparticles include the change in the color of gold, which at the macroscopic level is golden in color. However, in the case of nanoparticles of this material, the color will be dependent on the size, going from a reddish coloration to a blue color [50].

The area of medicine is one of the most demanding towards the use of materials with specific applications since the properties of these materials can be used in the development of drug delivery or transport, regenerative medicine, teragnostics, neural interfaces, biosensors, imaging, diagnostics, antibacterials, antivirals, and anticancer agents.

The transport mechanisms of nanoparticles in the human body are divided into active transport and passive transport. Nanoparticles are loaded with specific drugs. Nanoparticles can be recognized by some of the specific receptors of the cells, which allows them to bind to the cell membranes and penetrate into the interior of the cell to release the drug [51]. Passive transport nanoparticles are designed to be absorbed specifically by target cells without the use of any binder. Usually, nanoparticles must have specific sizes, shapes, and charges that, among other characteristics, direct transport to specific tissue areas via magnetic fields; in this case, functionalized magnetic nanoparticles are usually used, such as magnetite [52,53].

**Figure 1:**
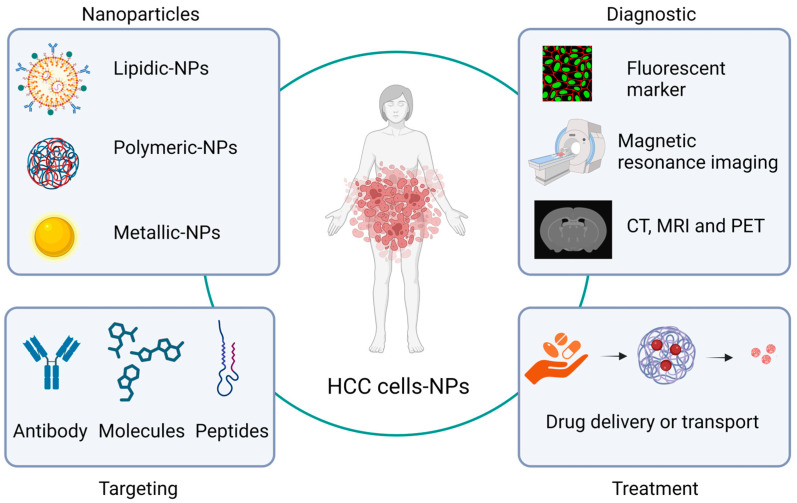
Key points in the medical applications of nanoparticles for HCC diagnosis and treatment. CT, computed tomography; MRI, magnetic resonance imaging; PET, positron emission tomography; NPs, nanoparticles.

In terms of HCC treatment, at least three of these nanoparticle applications in the medicine area—drug delivery, biomarkers, imaging, and diagnostic systems—are the most used. In relation to imaging and diagnostic systems, nanoparticles have been synthesized, directed toward HCC cells, or functionalized with some molecules (antibodies or peptides) related to this type of cell. This allows the nanoparticles to selectively bind to receptors or antigens present in HCC cells, either as contrast agents for magnetic resonance imaging analysis or as fluorescent markers, giving images high specificity [54]. In this regard, Ma X.H. et al. used iron oxide nanoparticles conjugated with double antibodies to target alpha-fetoprotein and glypican-3 (AFP and GPC3) antigens, which allows detection for the diagnosis of HCC [55]. On the other hand, Wang L. et al. used nanoliposomes, approximately 100 nm in diameter, coupled with anti-CD44 antibodies expressing doxorubicin or a triple fusion gene including herpes simplex virus truncated thymidine kinase (HSV-ttk), renilla luciferase (Rluc), and red fluorescent protein. These can be traced using the Rluc imaging technique, and it could be observed that nanoliposomes are accumulated on the surface of HCC cells. Nanoparticles containing doxorubicin also served to favor apoptosis in these cells [54] (Figure 1).

### 3.1. Biomarkers

The use of biomarkers in medicine is one of the most demanded branches of nanoscience because it allows the generation of rapid diagnostic methods, which are expected to be fast, cheap, and practical. One of the main problems with HCC is that, in the early stages, patients do not show symptoms that indicate the presence of this disease. Therefore, the development of highly sensitive sensors will allow for early detection in order to carry out an adequate treatment. In particular, the use of graphene sheets has been proposed, as one of their main properties is that their charge carriers have high mobility as well as a zero-energy gap, which allows them to be used as channels in field transistors (FET). This allows them to be used as analytical sensors [56]. Due to the above, Kim D.H. et al. proposed the detection of alpha-fetoprotein in plasma, which is often associated with the presence of HCC in adults, by developing a graphene-based sensor functionalized with 1-pyrenebutyric acid N-hydroxysuccinimide ester (PBASE) for the immobilization of an anti-AFP antibody that targets AFP. This causes changes in the Dirac point voltage (ΔVDirac), enabling the detection of AFP at a concentration of 0.1 ng/mL in PBS and 12.9 ng/mL in plasma, with detection sensitivities of 16.91 and 5.68 mV, respectively [57].

### 3.2. Theragnostic

Theragnostic is a branch that, according to Pene F. et al., is in charge of developing strategies for treatments that combine therapy with diagnosis in real-time [58]. This is one of the main applications of nanoparticles for the treatment of HCC, where they are proposed to function as markers and act against these cells. There are studies in vitro on the efficiency of mesoporous silica nanoparticles (MSNs), which have been used as carriers of anticancer drugs with hydrophobic properties. Experiments in which MSNs were functionalized with ruthenium polypyridyl complexes and with the Arg-Gly-Asp (RGD) peptide improved the cellular adsorption of the nanoparticles due to the presence of the peptide, which promoted cell adhesion and migration [59].

Another interesting study is the use of gold nanoparticles (AuNPs) for the diagnosis and treatment of HCC. Zhao Jun et al. applied silica-coated superparamagnetic iron oxide nanoparticles decorated with gold nanoparticles and loaded them into adipose-derived mesenchymal cells (AD-MSCs). The latter has the capacity to migrate to the liver area with lesions, so they can be used as transport vehicles in the organism, in this case, the superparamagnetic iron oxide-coated gold nanoparticles (SPIO@AuNPs system) [60]. Gold nanoparticles are used due to their magnetic nature to obtain magnetic resonance images as well as to ablate cancer cells because of the excitation of the gold nanoparticles by a near-infrared laser, which can increase the local temperature of the cells, generating cell death [61,62]. In recent times, the emergence of nanoscience has originated studies on the use of the properties of nanoparticles in the area of medicine, which have created a new branch of research known as nanomedicine [63,64,65,66]. Nanomedicine is responsible for understanding the mechanisms by which nanoparticles act in the human body, as well as their release and toxicity. In particular, studies of nanoparticles for the treatment of different types of cancer are a promising area in the field of nanomedicine [67]. Several formulations have been employed specifically for HCC, which are mostly included in lipid-based nanoparticles, metallic nanoparticles, and polymeric nanoparticles. 

### 3.3. Classification of Nanoparticles

Nanoparticles can be classified into various categories based on their composition, size, shape, and surface properties. Here are a few common classifications of nanoparticles and their use in HCC. The classification of nanoparticles is a fundamental aspect of nanoscience and nanotechnology, allowing researchers to categorize these minute structures based on their distinct characteristics and properties. This classification aids in comprehending their behavior, interactions, and utilization in various fields, from materials science and medicine to electronics and environmental remediation. By understanding how nanoparticles are classified, we can better appreciate the diversity and versatility of these nanoscale entities and harness their potential for innovative applications that continue to shape our modern world.

#### 3.3.1. Organic Nanoparticles

Organic nanoparticles are composed of organic materials such as lipids, proteins, or polymers. They are commonly used for drug delivery in HCC treatment, where drugs are encapsulated within the nanoparticles and then selectively delivered to tumor cells, improving their effectiveness while reducing systemic side effects [68].

#### 3.3.2. Inorganic Nanoparticles

Inorganic nanoparticles are typically made of inorganic materials like metals, metal oxides, or quantum dots. In HCC, inorganic nanoparticles can be used for various applications, including targeted imaging, photothermal therapy, and drug delivery. For example, gold nanoparticles can be functionalized with specific ligands to selectively target tumor cells, and when exposed to near-infrared light, they produce localized heat that can destroy cancer cells. Magnetic nanoparticles contain magnetic materials like iron oxide or magnetite. They can be used for targeted drug delivery and imaging in HCC. By applying an external magnetic field, magnetic nanoparticles can be directed to the tumor site, enhancing their accumulation and retention within the tumor [69].

#### 3.3.3. Carbon-Based Nanoparticles

**Figure 2:**
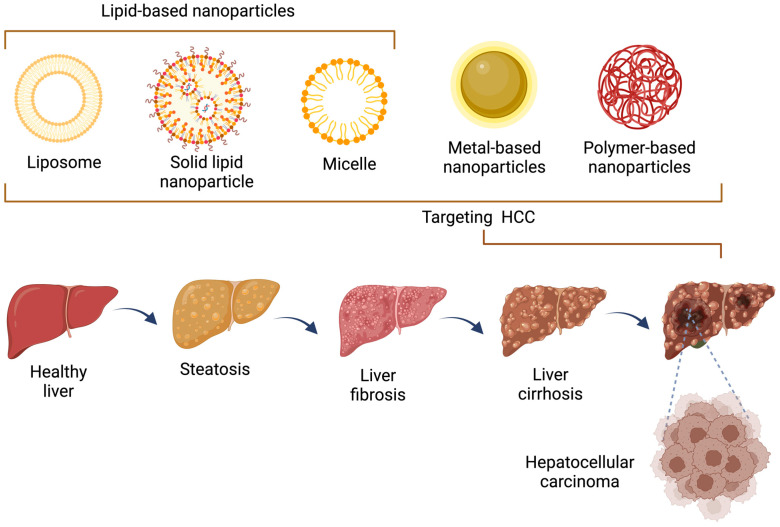
Different types of nanoparticle-mediated strategies for HCC therapy. HCC, hepatocellular carcinoma.

Carbon-based nanoparticles have unique properties that make them suitable for various applications in HCC. They can be used for targeted drug delivery, hyperthermia therapy, or photodynamic therapy. Carbon-based nanoparticles can also be functionalized with tumor-targeting ligands or fluorescent dyes for diagnostic imaging [70]. Hybrid nanoparticles are composed of multiple materials, combining the advantages of different nanoparticle types. For instance, a hybrid nanoparticle may consist of an organic shell for drug encapsulation and an inorganic core for imaging or magnetic targeting, providing versatility, and it can be tailored to specific requirements in HCC treatment [71]. The use of nanoparticles in HCC offers several benefits, including improved drug targeting and efficacy, enhanced imaging capabilities, and reduced systemic toxicity. However, it is important to note that the specific use and effectiveness of nanoparticles in HCC may differ depending on the specific nanoparticle design, the stage of the disease, and individual patient characteristics. Based on the wide ramifications of nanotechnology applications, this review provides some of the most outstanding NPs used in the treatment and diagnosis of HCC, focusing on the key results obtained using this type of nanomaterial (Figure 2).

## 4. Nanoparticles for the Diagnosis and Treatment of HCC

### 4.1. Liposomes

Liposomes are vesicles made of phospholipids stacked in concentric layers that are created by a lipid bilayer and are typically employed to encapsulate some aqueous phase. Emulsions are formed from a single layer of some surfactant, and they are usually used to encapsulate a liquid within another liquid. Both types of structures are frequently used for the transport and release of a drug because the size of these structures allows them to easily penetrate the skin or cell membranes [72].

#### 4.1.1. Diagnostic Application of Liposomes for HCC

Liposomes are usually functionalized with specific antibodies, peptides, proteins, and nucleic acids. Examples of such molecules are developed against tumor antigens such as alpha-fetoprotein (AFP) and des-gamma-carboxy prothrombin (DCP) or peptides such as arginylglycylaspartic acid (RGD). At the same time, liposomes encapsulate some contrast agents, such as gadolinium and indocyanine green, among others. Both the size and specificity of these types of systems allow for early and timely detection of various types of cancer, including HCC.

In particular, J. Zhou et al. conducted studies on the transfection of the ferritin heavy chain gene (FTH), which is reported to promote HCC cell proliferation and make HCC cells resistant to ferroptosis. For this purpose, the AFP promoter with FTH (AFP-FTH) has been modified, which allows the detection of HCC [73]. It performed the synthesis of nanoliposomes, with average sizes of 92 nm, obtained from 1,2-distearoyl-sn-glycero-3-phosphoethanolamine PEG Maleimide (DSPE-PEG-Tt) by the ultrasound method, previously reported by X. Li, in which two models were studied, one with transferrin (TF) as a ligand and the other in a pristine state (without ligand), which were labeled with indocyanine green (ICG). The liposomal systems were injected intravenously into mice, and fluorescence images of major organs were obtained, showing a higher accumulation in the liver and in tumor areas. The fluorescence of the liposomal system with TF and ICG showed a significantly more intense signal compared to that when no TF was added [74].

It has been reported that reactive oxygen species (ROS) are elevated in tumor areas. Therefore, Vera S. Shashkovskaya et al. used lipid nanoparticles (LNPs) of approximately 110 and 170 nm, loaded with pro-fluorescent dye (hydrocyanine-5) or plasmid DNA (pDNA), with genetically encoded protein sensors of hydrogen peroxide (HyPer7). This delivery system of fluorescent probes was aimed at obtaining visualization of ROS in the mouse xenograft model of HCC.

Hydro-Cy5-LNPs were injected into mice intravenously, and by fluorescence tests, it was determined that particles smaller than 110 nm accumulated mainly in the liver, while those of 170 nm were deposited in the spleen. In addition, it was observed that the nanoparticles transfected the liver from 6 to 14 days without the need for reinjection. On the other hand, they demonstrated the successful delivery of pDNA and hydro-Cy5 encapsulated in the lipid nanoparticles. Moreover, the accumulation of hydro-Cy5-LNPs allows visualization of the ROS zones of the tumor tissue in a mouse model [75].

#### 4.1.2. Therapeutic Application of Liposomes for HCC

In addition, liposomes can be functionalized or labeled to release drugs at specific sites and perform this release in a controlled manner. Doxorubicin encapsulation in liposomes has been extensively studied for the treatment of HCC. Doxorubicin is a chemotherapy drug that is commonly used for the treatment of different types of cancer, including HCC [76]. Doxorubicin acts by interfering with the DNA of cancer cells, preventing cell growth and division. Doxorubicin is also known to intercalate into DNA, inhibiting both DNA and RNA polymerase and ultimately ceasing DNA replication and RNA transcription [77]. There are some side effects (hair loss, nausea, fatigue, decreased appetite, and it may have toxic effects on the heart), so it is not usually recommended for patients with heart disease. The reduction in the side effects of this drug has been achieved by encapsulating it in liposomes, which reduces the side effects in normal cells [64]. Therefore, Allen T.M. and Martin F.J. have proposed the use of STEALTH, which consists of a liposomal system coated with polyethylene glycol, which helps the absorption of the liposomal system occur more slowly than the uncoated system in the reticuloendothelial system (RES), which improves the drug release in the areas where the tumor is located [78].

The RES refers to the set of cells and immune tissue, mostly located in the liver, that are capable of phagocytizing foreign materials and particles, as well as participating in cytotoxicity against tumor cells and regulating the immune system [79]. Nanoparticles have the potential to be absorbed by the RES; therefore, they are frequently coated with particles that mimic particular cell membrane regions or have hydrophilic qualities in order to “fool” the RES [80]. Regarding this type of system for the treatment of HCC, Fan YP et al. reported the synthesis of Folate-PEG-bis-amine and cholesterol-OTs (Chol-OTs) nanoliposomes according to the methodology reported by Yang T. et al., where they are loaded with doxorubicin and L-miR-375/doxorubicin [81,82]. miRNA-375 is involved in the regulation of some cellular processes, including cell proliferation, apoptosis, and tumor development. Fan YP et al. investigated that this combination not only inhibits the malignant phenotypes of HCC cells but also acts as a tumor suppressor in vitro and in vivo models. Furthermore, they report that dosing doxorubicin in liposomal form decreases resistance to doxorubicin due to the downregulation of MDR1 expression by targeting AEG-1 [81]. On the other hand, Cannito S. et al. prepared nanolipids with diameters between 165 and 225 nm using 1,2-distearoyl-sn-glycero-3-phosphocholine (DSPC), cholesterol (CHOL), and 1,2-distearoyl-sn-glycero-3-phosphoethanolamine-N-[amino(polyethylene glycol)-2000] (mPEG2000-DSPE, PEG) (55:40:2 molar ratio) or only DSPC/CHOL [83]. These were decorated with hyaluronic acid (HA) because it binds to the N-terminal CD44 protein, which has been observed to be increased in patients with HCC. The results showed that the nanoliposomes were internalized by the Huh7 cell line, which is derived from HCC and overexpresses CD44. In addition, they observed that in the murine and human macrophage cell models, uptake was enhanced without the participation of CD44. These results are interesting because they show that this model can target tumor and liver cancer cells, which can counteract the progression of HCC [54,84].

### 4.2. Solid Lipid Nanoparticles

Solid lipid nanoparticles (SLNs) are aqueous colloidal dispersions with a solid biodegradable lipid matrix. SLNs combine the advantages and avoid the disadvantages of a few colloidal carriers, for example, physical stability, protection assurance of fused labile medications from protection, assurance of incorporated labile medications controlled release, and excellent tolerability [85].

#### 4.2.1. Diagnostic Application of Solid Lipid Nanoparticles for HCC

Solid lipid nanoparticles are systems that are usually used to transport drugs into the body; however, it is possible to take advantage of their properties to use them as carriers of contrast agents or imaging agents to distinguish different tumor areas, including HCC. However, most of the studies that can be found in the specialized literature are based on the use of this type of nanoparticle mainly as drug carriers and leave in the background their capacity to carry contrast agents. A study carried out by A. Grillone et al. used sorafenib together with superparamagnetic iron oxide nanoparticles (SPIONs) encapsulated in SLNs obtained from cetyl palmitate. Their results showed that this type of structure had the ability to inhibit the proliferation of cancer cells due to the cytotoxic action of sorafenib. In addition, it was observed that there was a strong uptake of the nanoparticles by HepG2 cells. Additionally, being magnetic nanoparticle carriers opens the possibility of following the particle’s path because they have a magnetic moment. This could be proven by comparing this system with several commercial contrast particle systems, such as Endorem, Revosit, and Sinerem, which also use SPION. Furthermore, this type of system can be used as a negative contrast agent to track its use in magnetic resonance imaging (MRI) [86].

#### 4.2.2. Therapeutic Application of Solid Lipid Nanoparticles for HCC

In vitro and in vivo studies have been conducted to create and characterize SLN formulations for various application routes (parenteral, oral, dermal, ocular, pulmonary, and rectal) [87]. Lipids can be triglycerides (tri-stearin), partial glycerides, fatty acids (stearic acid), steroids, and waxes (cetyl palmitate). Various emulsifiers and their combinations have been used to stabilize lipid dispersion [88]. Mahfoozur Rahman et al. focused on the creation and evaluation of resveratrol (RV)-loaded cationic solid lipid nanoparticles (RV-c-SLNs) for hepatocellular carcinoma management. RV-c-SLN significantly reduced the tumor volume and increased its accumulation in tumor tissue in rats compared to RV solution and RV-SLN. Furthermore, RV-c-SLN reduced the levels of pro-inflammatory cytokines (TNF, IL-1, IL-6, and NF-kB) while balancing antioxidant enzymes (SOD, CAT, GPx, and GSH). Histopathological examination revealed a reduction in the occurrence of hepatic nodules, necrosis development, inflammatory cell infiltration, blood vessel inflammation, and cell swelling [89].

Yumeng Wei et al. (2022) studied long-circulating solid lipid nanoparticles (LSLN) containing a novel curcumin derivative (CU1) in order to improve the pharmacokinetic behavior of curcumin derivative-loaded solid lipid nanoparticles (CU1-LSLN) and their anticancer effects in MHCC-97H liver cancer cells. After 3 h of treatment, CU1-LSLN absorption efficiency in MHCC-97H cells was higher than that of curcumin and CU1 (3.24 and 2.98 times, respectively), which contributed to CU1-LSLN’s inhibitive effect on MHCC-97H cell proliferation, migration, and invasion and increased its ability to promote apoptosis. Meanwhile, the expression of NF-kB, COX-2, MMP-2, MMP-9, and uPA decreased considerably. Because of LSLN’s distinct metabolic activity, CU1-LSLN has a significantly higher relative bioavailability and improved pharmacokinetic behavior [90].

### 4.3. Micelles or Nanoemulsions

Micelles are a type of self-assembled nanostructure that forms in certain solutions. They consist of a spherical arrangement of amphiphilic molecules, which have both hydrophilic (water-loving) and hydrophobic (water-fearing) regions. The hydrophilic regions face outward, interacting with the surrounding water, while the hydrophobic regions cluster together at the core of the micelle, away from the water.

#### 4.3.1. Diagnostic Application of Micelles or Nanoemulsions for HCC

The use of nanoemulsions for HCC detection is one of the applications of nanotechnology in the medical field. Although its main focus is on the release of drugs in specific or localized areas, in recent years, applications have been proposed in which contrast agents, such as quantum dots, magnetic nanoparticles, or fluorescent molecules, are usually encapsulated. This allows for overcoming some limitations that some materials with low solubility may have. In addition, their surface can be functionalized with specific molecules, allowing them to be targeted at specific sites. Some applications of this type of system are found in the research carried out by J. Zhu et al., who encapsulated indiocyanine green (ICG) and lipiodol in tween 80 and lecithin via ultrasound methods, obtaining an average hydrodynamic radius of 60 nm. They observed that the nanoemulsified ICG absorbance showed a redshift, presenting an absorbance at 805 nm and an improvement in intensity. The authors proposed to use these particles as fluorescent contrast agents, which provide better visualization of tumor margins and, thus, better resection of tumor tissue. Their results showed that the fluorescence intensity remained higher than that observed in ICG without emulsification. When an 808 nm laser was applied, its intensity decreased by almost 50%, while for the emulsified sample, it remained at 80%. In addition, after 60 min, a fluorescence signal still remains for the system proposed by the authors, while for ICG alone, this signal was lost. In their analysis using HepG2, they showed that after 8 h of incubation, there was a higher uptake of the emulsified system (68%) compared to the pristine system (32%) [91]. In addition to the above, when scanning by laser confocal microscopy, it was observed that the samples treated with the nanoemulsions showed fluorescence in the cytoplasm, while the response was almost nil for the unemulsified system. On the other hand, the nanoemulsions were injected into orthotopic mice with hepatic tumors, which were operated on 24 h after the injection. Luminescence imaging was used as a guide for tumor resection. Subsequent analysis of the tissue showed that the tumor had been completely removed, thanks to the fact that this methodology allows the visualization of small tumors as well as the edges of the tumors. Another group, N. Anton et al., performed the encapsulation of 2-3-5-triiodo α-tocopheryl in PEGylated nanoemulsions, which helps to prevent renal clearance and thus prolong the time in the blood by 9 h. Their results showed an outstanding contrast in the liver within 3 months after the injection. Studies in mouse models of HCC tumors using microcomputed tomography (microCT) showed that the injection of the nanoemulsions allowed an increase in the liver contrast due to the affinity of the droplets towards the liver cells. However, it was noted that they induced negative contrast in the cells immersed in the tumor. The use of nanoemulsions makes it possible to encapsulate contrast agents that can be used in different techniques, thus allowing precise determination of tumor areas in a simple and effective manner [92].

#### 4.3.2. Therapeutic Application of Micelles or Nanoemulsions for HCC

Micelles or nanoemulsions are commonly used in nanotechnology and various other fields due to their unique properties and structure [93]. Micelles have garnered significant attention due to their potential applications in drug delivery, cosmetics, and surface cleaning, among others. Regarding drug delivery, micelles can encapsulate hydrophobic drugs in their core, protecting them from degradation and allowing them to be targeted for delivery to specific tissues or cells. Additionally, their small size (typically tens to hundreds of nanometers) enables them to easily penetrate cell membranes [94]. In nanotechnology, micelles have been utilized as templates for the synthesis of nanoparticles, where the core of the micelle acts as a confined reaction space for the formation of nanoparticles with controlled size and composition [95]. A study by Zou J. et al. focused on the use of micelles for delivering ursolic acid and TRAIL (TNF-related apoptosis-inducing ligand) genes to HCC cells. This study demonstrated that the co-delivery of these therapeutic agents using micelles enhances their anti-HCC activity and increases apoptosis in cancer cells [96]. Another study by Le et al. performed the preparation and characterization of gemcitabine-loaded chitosan–PEG micelles [97]. In general, various nanoemulsions and nanoliposomes have been reported for HCC under different models (Table 1).

### 4.4. Polymer-Based Nanoparticles

Currently, the use of polymer-based nanoparticles for the treatment of HCC is one of the most required lines because they are generally biocompatible, allowing the encapsulation and controlled release of drugs or genes. In addition, it is possible to functionalize their surface with antibodies for targeted release to occur. Some of the most commonly used polymers in this line are polylactic acid, polyethylene glycol, and chitosan.

#### 4.4.1. Diagnostic Application of Polymeric Nanoparticles for HCC

In the detection of HCC through the use of polymer-based nanomaterials, they are usually used as encapsulation materials that can function as contrast agents, taking advantage of their properties such as low toxicity as well as their biocompatibility. Z. Karahaliloglu et al. performed the encapsulation of iron oxide nanoparticles with polyethylene glycol (PEG)-terminated polystyrene (PS)-linoleic copolymer (polyethylene glycol (PEG)-terminated polystyrene (PS)-linoleic copolymer), forming a core–shell structure. These structures showed a concentration-dependent hydrodynamic radius of polymer with respect to the nanoparticles used during the core–shell structuring process, which ranged from 148 to 202 nm. To confirm the uptake of the coated nanoparticles, studies were performed on mouse hepatocytes and fibroblasts using the Prussian blue assay. The nanoparticles were directed into the cytoplasm and perinuclear region of the cells, and they tended to internalize in cancer cells. On the other hand, they performed a phantom MRI test; that is, they suspended the coated nanoparticles in water and performed the analysis on them, finding that this model could give a good response for this type of analysis [98].

On the other hand, polymeric nanoparticles of poly(beta-amino-ester) (PBAE 536) loaded with DNA labeled with a fluorescent marker were administered to mice intravenously. The orthotopic xenograft model of HCC involves implanting Hep3b cells into the liver of NU/J athymic mice. The nanoparticles accumulated in the liver region, observing that a was in the 7% region of the fluorescence signal in the tumor region [99].

#### 4.4.2. Therapeutic Application of Polymer-Based Nanoparticles for HCC

Chitosan is a polysaccharide usually obtained from the deacetylation of chitin, the second most abundant biopolymer in nature. It is found in the exoskeleton of crustaceans, insect cuticles, and fungal cell walls. However, it can be naturally extracted from the walls of some types of fungi (Mucoraceae) [100]. Its properties are dependent on the degree of deacetylation and molecular weight. The main characteristic of chitosan is that it is only soluble in acidic media. Chitosan has antimicrobial, antitumor, and immunostimulant properties; however, antimicrobial is the most studied [101]. In addition, according to the study of Hari Sharan Adhikari and Paras Nath Yadav, chitosan can alter the normal mechanism of the cell cycle with enzymatic synthesis and interrupt the hormonal mechanism towards biosynthesis to inhibit the growth of cancer cells. They identified that chitosan can selectively penetrate through the membrane of cancer cells and generate apoptotic activity [102]. The effect of *Artemia salina* inhibitory properties on HCC using chitosan nanoparticles in vitro (HepG2) and in vivo (rats) has been performed by Mai M. Elkeiyi et al. [103].

Nanoparticles were synthesized by the ionic gelation method using tripolyphosphate as a crosslinking agent, with a size observed by the transmission electron microscopy technique of 50 to 70 nm. MTT assays to detect the inhibitory effect on HepG2 cells showed at 48 h a dose-dependent cytotoxic effect depending on the number of chitosan nanoparticles (100, 50, 25, 12.5, 6.25, and 3.125 μg/mL), with an IC_50_ of 25.2 ± 1.15 compared to untreated HepG2 cells. This effect was attributed to the nanoparticles increasing necrotic cells because they observed a positive regulation of RP1 expression with no significant change in the level of Bax (Bcl-2-associated X protein) or AIF (apoptosis-inducing factor) expression, so there was no increase in apoptosis [104]. However, because of its biodegradability, chitosan has been advocated for the therapy of HCC in several publications linked to HCC research.

In particular, simvastatin (SV) has been proposed as one of the drugs that are encapsulated in chitosan nanoparticles; it has been reported to inhibit tumor cell growth and anchor in the endothelium, which generates a decrease in the expression of β1, β3, and α2 integrins. For this purpose, chitosan and SV nanoparticles were formed using the ionic gelation method using TPP and chondroitin sulfate as crosslinkers (SVCSChSNPs). The average hydrodynamic diameters of the nanoparticles obtained showed an oval shape and diameters between 20 and 40 nm, as observed by transmission electron microscopy [105].

The outcomes of assays in HepG2 cells exhibited that the samples treated with IC50 showed 21.2% and 33.34% of early and late apoptosis, respectively, while for the control system, they were only 3.73% and 3.93%. These results are related to the size and positive charge of the particles, which increase the accumulation of SV in the intracellular compartments and increase cytotoxicity [106]. In general, certain polymeric nanoparticles for HCC have been documented in various models (Table 2).

**Table 1:** Main studies in vitro and in vivo of lipid-based nanoparticles.

Lipid-Based Nanoparticles					
Nanoformulation	Particle Size	Model	Drug Administration (Concentration/Dose)	Endpoints	Reference
Diosmin-loaded solid lipid nanoparticles (Diosmin-SLN)	37.48 nm	HepG2 cells In vivo induction HCC Wistar rats	In vitro: 0 to 40 µM of Diosmin-SLN In vivo: 5 mL/kg/day (25 mg/kg) via oral of Diosmin-SLN for up to 14 weeks	In vitro: ↑ Cytotoxicity ↑ Lipid peroxidation In vivo: ↓ Hepatic nodules ↑ Antioxidant parameters (SOD, GPx)	[107]
Celastrol-galactosylated Liposomes (C-GPL)	139.4 ± 2.7 nm	HepG2 cells Wild-type FVB/N mice	In vitro: 0.2 μg/mL of celastrol concentration In vivo: 2 mg/kg of C-GPL once every other day for 2 weeks.	In vitro: ↑ Cytotoxicity ↑ Apoptosis In vivo: ↓AST, ALT, AFP Suppression of AKT activation Retardation of cell proliferation	[108]
PEGylated paclitaxel-loaded galactosylated liposomes (Tn-Lipo-PTX)	74 ± 0.36 nm	HepG2 cells	In vitro: 50 ng/mL of PTX	Tn-Lipo-PTX exhibited enhanced cytotoxicity than free PTX Improved cellular uptake level based on EPR effect. ↑ CDK1 and cyclin B1 protein expression	[109]
Nanoliposome C6-Ceramide in combination with antiCTLA4 antibody	87 nm	In vivo induction HCC Male C57BL/6 mice	35 mg/kg body weight injection IV every other day at 200 μL volume for 2 weeks	↓ Tumor weight (g) ↓ KLF2, FoxP3 and CTLA4 expression	[110]

AST, aspartate aminotransferase; ALT, alanine aminotransferase; AFP, alpha-fetoprotein; AKT, protein kinase B; SOD, superoxide dismutase; GPx, glutathione peroxidase; CDK1, cyclin-dependent kinase 1; KLF2, Krüppel-like Factor 2; CTLA4, cytotoxic T-lymphocyte associated protein 4. ↑ increase in parameters; ↓ decrease in parameters.

**Table 2:** Relevant in vitro and in vivo revisions of polymer-based nanoparticles.

Polymeric Nanoparticles					
Nanoformulation	Particle Size	Model	Drug Administration (Concentration/Dose)	Endpoints	Reference
Simvastatin–chitosan nanoparticles co-crosslinked with tripolyphosphate and chondroitin sulfate (SVCSChSNPs)	<100 nm	HepG2 cells In vivo induction HCC Wistar albino rats	In vitro: SVCSChSNPs: 0.5, 1, 2.5, 5, 10, 25, 75, 100, 125, 150, 175, and 200 µg. In vivo pharmacokinetic: Single dose of 0.5 mL of simvastatin (SVCSChSNPs) dispersed in sterile water (equivalent to 20 mg/kg body weight) via oral gavage.	In vitro: ↑ Cytotoxicity (inhibited growth in HepG2 cells). ↑ Apoptosis In vivo: Improved oral bioavailability of the simvastatin High rate of cellular absorption via ASGPR-mediated endocytosis	[111]
Curcumin–polyamidoamine dendrimer	56.8 ± 21.5 nm	Huh-7, Hepa1-6 In vivo induction HCC BALB/c mice	In vitro: 5 to 45 μM (selected concentration 25 μM) In vivo: IP injection of 0.5 mg curcumin–polyamidoamine dendrimer (curcumin) per 25 g of body weight in 500 μL of PBS every day for 1 week, followed by a similar dosage on alternate days for the next 8 days.	In vitro: ↑ Cytotoxicity. ↑Apoptosis Cell cycle arrest at G2/M. ↑ROS ↑ Lipid peroxidation In vivo: ↓ Tumor weight (g). ↑Survival Rate in Mice	[112]
Curcumin derivative-galactosamine targeted G4 PAMAM dendrimers (PAMAM-GAL)	48.7 ± 7.03 nm	nu/nu female mice HepG2 cells HCC xenograft model	In vitro: 0.5 to 50 µM of PAMAM-GAL In vivo: 10 nmole/mice of PAMAM-GAL symmetrical near-infrared dye (S0456) via tail vein injection in the dye conjugate.	↑ Cytotoxicity High cellular uptake via ASGPR-mediated endocytosis. Improve the delivery of CDF into HCC cell lines.	[113]
Sorafenib-loaded dendritic polymeric nanoparticle (NP-TPGS-SFB)	118.3 nm	HepG2 HCC xenograft-bearing nude mice	In vitro: 0.1 to 12.5 μg/mL of NP-TPGS-SFB In vivo: 5 mg/kg of sorafenib (NP-TPGS-SFB) via the tail vein three times on days 0, 4, and 8 during 14 days of treatment.	In vitro: ↑Cytotoxicity In vivo: ↓ Tumor weight (g)	[114]

ASGPR, asialoglycoprotein receptor; ROS, reactive oxygen species; CDF, curcumin derivate; HCC, hepatocellular carcinoma; IP, intraperitoneal; PBS, phosphate-buffered saline. ↑ increase in parameters; ↓ decrease in parameters.

### 4.5. Metal-Based Nanoparticles

Metallic nanoparticles present unusual optical, magnetic, and thermal properties, which allow them to be used for the transport and controlled release of drugs, as well as in photothermal therapies and nuclear magnetic resonance imaging [66,115].

#### 4.5.1. Diagnostic Application of Metal-Based Nanoparticles for HCC

The optical and magnetic properties of metal-based nanoparticles are often used as contrast agents for medical imaging, nuclear magnetic resonance, and positron emission tomography. Metal-based nanoparticles have been used in the detection of HCC-specific biomarkers in both tissue and blood samples. In addition, these types of nanostructures tend to have high physical and chemical stability compared to other markers. They also have a high specific surface area, which can be functionalized with specific markers associated with HCC in order to increase their specificity and sensitivity, which helps in the timely and early detection of this disease.

Gold nanoparticles have been shown to have a high capacity to bind to proteins and lipids located on the surface of cancer cells. In particular, there is a wide range of possible metallic nanoparticles that can be used in this type of study, with some of the most studied nanostructures being those of gold and iron. Gold nanoparticle studies carried out by K. Mintz et al. showed the coupling of gold nanoparticles functionalized with polyclonal antibodies and carbon dots conjugated to a detection monoclonal antibody in order to increase the optical response of those cells that had alpha-L-fucosidase (AFU) present. There are some reports showing the presence of elevated serum levels of AFU in patients with cirrhosis and HCC. Their results showed that they were efficient in detecting cells with AFU in both peripheral blood smears (PBSs) and blood. In addition, it was observed that the use of carbon quantum dots allowed an increase in detection efficiency when compared to the commonly used fluorescein isothiocyanate (FITC), which is normally used to stain proteins and nucleic acids. The detection improvement using the gold nanoparticles and carbon quantum dots system compared to the gold nanoparticles and fluorescein isothiocyanate system is due to the fact that there is a greater overlap in the absorbance spectrum between the gold nanoparticles and the carbon dots than with the gold nanoparticles and fluorescein isothiocyanate system [116].

Another example of nanoparticles used for the detection of HCC are iron nanoparticles, which usually present a magnetic behavior that allows them to interact with external magnetic fields, making them suitable for their application in magnetic resonance analysis. These particles present an additional advantage that has been observed to play a role in biological systems. Studies by XH Ma et al. have shown that surface functionalization of ultra-small superparamagnetic iron oxide nanoparticles (USPIO), with an average size of 5 nm, with two types of biomarkers, alpha-fetoprotein (AFP) and glypican 3 (GPC3), allows increasing specificity in the diagnosis of HCC. Usually, only a single biomarker is used for this type of study, and they can detect tumors smaller than 1 cm. The murine hepatoma cell line (Hepa1-6) was used for their assays.

Results showed a 23.3% absorption of the system with the two antibodies anti-AFP-USPIO-anti-GPC3 (UAG) compared to anti-AFP–USPIO (UA). Comparatively to the unlabeled USPIO, the single antibody-conjugated USPIOs also demonstrated better cell binding efficiency. In the MRI analysis, a reduction in the T2 relaxation time was observed in UAG-treated cell samples compared to UA, anti-GPC3–USPIO (UG), and unlabeled USPIO. The reductions were 14.93, 9.38, and 15.3%, respectively, which favors the study of the images obtained by this technique since it allows for better distinguishing the different tissues in an image [54,117].

#### 4.5.2. Therapeutic Application of Metal-Based Nanoparticles for HCC

The routes of administration of nanoparticles can range from intravenous injection, inhalation, ingestion, or topically, depending on the area of the organism where they are put to act. In particular, gold is considered a good transport element because it is an inert material that does not react chemically in the body and can be considered biocompatible [118]. It also has optical properties that allow it to be used in photothermal ablation processes. In this regard, Mocan L. et al. have performed studies on systems composed of gold nanoparticles with albumin with an average diameter of 48 nm. The binding between the surface of the nanoparticles and albumin is carried out by exploiting the disulfide groups present on the protein. The choice of functionalization with albumin was made based on studies in which it has been observed that malignant tumors tend to process large amounts of this protein in the lysosomal compartment of the cells. The composite system was found to be taken up by HepG2 cells at a higher rate than either nanoparticles or albumin alone. To ensure composite system selectivity, they selected the left hepatic lobe from three patients with HCC showing a hypoechoic round node. A laser beam with a wavelength of 808 nm and a power of 5 W/cm^2^ was applied to the liver 1 to 2 cm above the tumor and irradiated for 30 min. Their results showed that the treatment allows the generation of selective cell death due to the DNA damage produced by the thermal changes caused by the excitation of the electronic cloud of the nanoparticles generated by the laser beam [119].

In the studies conducted by Hui-Ying Xue on the treatment of HCC, gold nanoparticles with a hydrodynamic radius of approximately 36 nm were functionalized with a miR-375 mimic and PEG to stabilize them. As a result, NPs reached a fine hydrodynamic radius averaging 53 nm. MiR-375 is present in animal cells and has been shown to act as a tumor suppressor of HCC because it inhibits P-gp expression through inhibition of AEG-1 and induces apoptosis by targeting AEG-1 and YAP1 [120]. The binding of the gene and the nanoparticle is carried out through the formation of a covalent bond between the nanoparticle and the sulfur atoms in the gene. In vitro studies showed growth inhibition and death in HepG2 and Hep3B cells, as well as dose-dependent behavior. To study whether the binding gene-nanoparticle binding did not affect the functions of miR-375, the authors determined the expressions of this gene in Hep3B cells when treated with Au-miR-375 using western blot, and they observed its three main targets, which are AEG-1, YAP1, and ATG7, in HCC, finding a significant reduction in these genes [120]. Several metallic nanoparticles have been reported for HCC in different models (Table 3).

## 5. Nanoparticles-Based Drug Delivery for Hepatocellular Carcinoma

**Figure 3:**
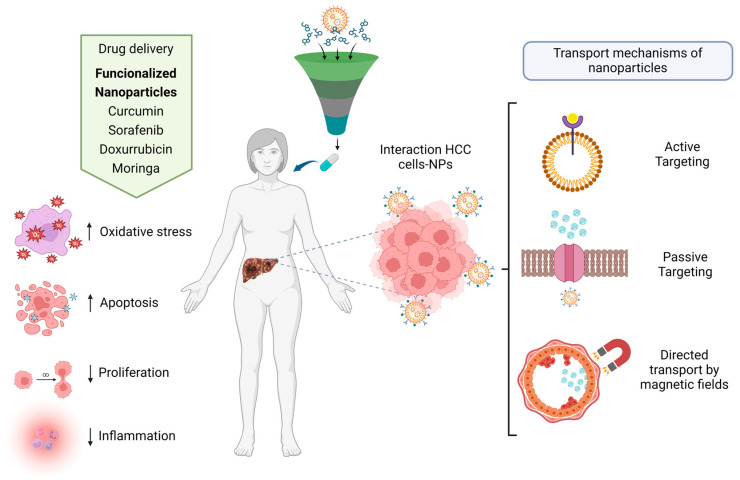
Drug delivery of nanoparticles functionalizing with drugs–therapy of HCC, and types of transport mechanisms of nanoparticles.

The properties and synthesis processes of distinct nanomaterials, whereby optimal nanocarriers are produced and designed, determine the shape, size, structure, and transport functions of nanoparticle-based drug delivery systems. Drug delivery systems have made great advancements in the targeted therapy of liver cancer. They have demonstrated efficacy in animal models and human studies, showing their extensive application possibilities in cancer treatment. Some NPs have demonstrated good liver targeting, slow-release, and modifiability, which can boost medication concentrations in the liver, improve treatment efficacy, and reduce drug toxicity and adverse effects [125,126] (Figure 3).

Several nanoparticle-based therapies have been explored in clinical trials throughout the last decade. Table 4 lists the clinical trials using nanoparticle-based drug delivery and therapy registered on the ClinicalTrials.gov web site (July 2023) for the treatment of hepatocellular cancer.

## 6. Conclusions and Future Perspectives

In conclusion, there have been significant advances in the diagnosis and treatment of hepatocellular carcinoma through the application of nanotechnology. Firstly, nanotechnology has greatly improved the early detection and diagnosis of hepatocellular carcinoma. Nanoparticles can be designed to specifically target cancer cells and deliver improved imaging agents, allowing highly sensitive and accurate imaging techniques such as magnetic resonance imaging (MRI) or positron emission tomography (PET) to detect even small tumors. This early detection enables prompt intervention and improves patient positive outcomes. Secondly, nanotechnology has transformed the treatment options for hepatocellular carcinoma. Since the FDA approved the clinical application of organic microspheres, like liposomes, alongside well-studied iron oxide NPs, nanomaterials have seen advancements in terms of biocompatibility, biodegradability, diameter and size, and specific physico–chemical properties. These advancements allow the development of novel strategies for HCC treatment, like thermal ablation, chemoembolization, and immunotherapy. However, manufactured nanoparticles have many challenges, including the toxicity of materials, possible environmental hazards, reduced production costs, stability issues after scale-up for manufacturing, and a lack of agreed-upon regulations for the use, disposal, and recycling of nanomaterials, to name a few. In clinical scenarios, the propensity of NPs to accumulate in lymphoid organs when administered systemically is well known. Also, the preferential accumulation in the kidneys of some polymer-bound drugs could lead to nephrotoxicity, and unexpected toxicity can also result from excipients. Moreover, the induction of pseudo-allergic responses associated with specific NPs in the literature, in addition to the immunotoxicological effects of certain NPs, requires further attention. Drug hemolysis, complement activation, cytokine release, opsonization, phagocytosis, and pharmacokinetics studies represent part of the data required for the clinical translation of nanomedicines. Specifically, for HCC applications, there is a need for high quantities of NPs and the best possible targeted and controlled release, some facts that are still uncertain for the application of nanotechnology. It is important to pay special attention to the known tumor-increased angiogenesis and its effect on NPs distortion or particle breakdown when passing through vascular wall cells and on NPs or its residue accumulation in tumor tissue that could lead to a possible thrombosis. The relationship between dose, bioprocessing, and HCC response is an area of active research, as all three may be related in a non-linear manner. Future studies are required where standardization of the therapeutics can be accomplished in patients with similar HCC stages, and the efficacy of nanomedicine should be monitored through application and at a later time to detect liver or renal toxicity, side effects, and sudden loss of effectiveness that could affect HCC therapy. However, with a multidisciplinary approach, NPs can evolve to be the solution to the actual needs of HCC patients, alone or in combination with other standard therapies.

## Figures and Tables

**Table 3:** Major studies in cells and animal models of metal-based nanoparticles.

Metallic Nanoparticles					
Nanoformulation	Particle Size	Model	Drug Administration (Concentration/Dose)	Endpoints	Reference
Curcumin–silver nanoparticles	12 ± 2 nm	In vivo induction HCC Male CD1 albino mice	High dose: 3.3 mg/kg orally by gavage twice/week for 15 weeks. Low dose: 0.6 mg/kg orally by gavage twice/week for 15 weeks.	↓ VEGF serum levels ↓ TNFα serum levels ↓ AFP serum levels ↓ NFκβ expression ↑ caspase-3 expression	[121]
Sorafenib–gold nanoparticle-loaded anti-miR-221 (AuNPs-anti-miR221)	13 nm	HepG2 and Huh7 cells	Synergistic effect Combination of Sorafenib: 0, 5, 10, 20 µM and AuNPs-anti-miR221: 0, 0.1, 0.2, 0.5, 1, 2, 4, 8 nmol/L	AuNPs-anti-miR-221 and sorafenib caused: ↓ miR-221/p27/DNMT1 signaling pathway. ↑ Apoptosis ↓ Proliferation cellular	[122]
Curcumin–selenium nanoparticles	53 nm	HepG2 cells and LO2 cells and HCC xenograft model (female nude mice)	3 mg/kg, 6 mg/kg, and 9 mg/kg IV injection every 2 days and continued for 16 days.	↓ Tumor weight (g) ↑ Apoptosis (ROS generation) ↓ Migration of HepG2 cells ↑ caspase-3 expression	[123]
*Moringa oleifera* leaves extract combined with vitamin C-functionalized selenium nanoparticles (MO/asc.-SeNPs)	30 nm	In vivo induction HCC Wistar albino rats	IP injection of MO/asc.-Se-NPs (1 mL/kg) (once a week)	↓ AST, ALT, ALB ↓ MDA ↓ IL-6 concentration (pg./mL)	[124]

HCC, hepatocellular carcinoma; IP, intraperitoneal; VEGF, vascular endothelial growth factor; TNFα, tumor necrosis factor-alpha; NFκβ, nuclear factor kappa B; miR-221, microRNA-221; DNMT1, DNA methyltransferase 1; ROS, reactive oxygen species; AST, aspartate aminotransferase; ALT, alanine aminotransferase; ALB, alkaline phosphatase; MDA, malondialdehyde; IL-6, interleukin 6. ↑ increase in parameters; ↓ decrease in parameters.

**Table 4:** Clinical trials using nanoparticle-based drug delivery and therapy for hepatocellular carcinoma.

NCT Number	Study Title	Conditions	Intervention	Study Type	Status/Phases
NCT02314052	Phase Ib/2, Multicenter, Dose Escalation Study of DCR-MYC in Patients With Hepatocellular Carcinoma	Hepatocellular Carcinoma, with at least one measurable lesion > 10 mm (excluding bone metastases)	Drug: Double-stranded RNA in a stable lipid particle suspension	Interventional	Completed Phase 1 and Phase 2
NCT04682847	Radiotherapy With Iron Oxide Nanoparticles (SPION) on MR-Linac for Primary and Metastatic Hepatic Cancers	Liver neoplasms, hepatic cirrhosis, hepatic carcinoma, liver cancer, liver metastases, liver carcinoma, hepatocellular carcinoma, hepatocellular cancer, and hepatic atrophy.	Drug: Iron Oxide Nanoparticles (SPION)	Observational	Active, not recruiting
NCT02716012	First-in-Human Safety, Tolerability and Antitumour Activity Study of MTL-CEBPA in Patients With Advanced Liver Cancer (OUTREACH)	Hepatocellular Carcinoma, Liver Cancer	Drug: Double-stranded RNA formulated into a liposomal nanoparticle and is designed to activate the CEBPA gene. Sorafenib 200 mg	Interventional	Active, not recruiting. Phase 1
NCT02181075	Targeted Chemotherapy Using Focused Ultrasound for Liver Tumours (TARDOX)	Liver Tumour	Drug: Lyso-thermosensitive liposomal doxorubicin	Interventional	Completed, Phase 1
NCT02721056	A Phase I-II Study of NBTXR3 Activated by Sterostatic Body Radiation Therapy (SBRT) In the Treatment of Liver Cancers	Liver cancer	Radiation: NBTXR3, IL or IA injection + SBRT	Interventional. Single Group Assignment	Completed, Phase 1 and 2
NCT00441376	A Phase I Dose Escalation Tolerability Study of ThermoDox (Thermally Sensitive Liposomal Doxorubicin) in Combination With Radiofrequency Ablation (RFA) of Primary and Metastatic Tumors of the Liver	Hepatocellular carcinoma Liver neoplasms	Drug: ThermoDox (Thermally Sensitive Liposomal Doxorubicin)	Interventional	Completed, Phase 1
NCT00617981	A Phase III, Randomized, Double-Blinded, Dummy-Controlled Study of the Efficacy and Safety of ThermoDox (Thermally Sensitive Liposomal Doxorubicin) in Combination With Radiofrequency Ablation (RFA) Compared to RFA-Alone in the Treatment of Non-Resectable Hepatocellular Carcinoma	Hepatocellular carcinoma	Drug: ThermoDox (Thermally Sensitive Liposomal Doxorubicin)	Interventional	Completed, Phase III
